# Bone mineral density over ten years after primary parathyroidectomy in multiple endocrine neoplasia type 1

**DOI:** 10.1093/jbmrpl/ziae129

**Published:** 2024-10-22

**Authors:** Emma Kuusela, Iiro Kostiainen, Elina Ritvonen, Eeva M Ryhänen, Camilla Schalin-Jäntti

**Affiliations:** Endocrinology, Abdominal Center, Helsinki University Hospital and University of Helsinki, ENDO-ERN (European Reference Network on Rare Endocrine Conditions), FIN-00290 Helsinki, Finland; Endocrinology, Abdominal Center, Helsinki University Hospital and University of Helsinki, ENDO-ERN (European Reference Network on Rare Endocrine Conditions), FIN-00290 Helsinki, Finland; Endocrinology, Abdominal Center, Helsinki University Hospital and University of Helsinki, ENDO-ERN (European Reference Network on Rare Endocrine Conditions), FIN-00290 Helsinki, Finland; Heart and Lung Center, Helsinki University Hospital and Helsinki University, FIN-00290 Helsinki, Finland; Endocrinology, Abdominal Center, Helsinki University Hospital and University of Helsinki, ENDO-ERN (European Reference Network on Rare Endocrine Conditions), FIN-00290 Helsinki, Finland; Endocrinology, Abdominal Center, Helsinki University Hospital and University of Helsinki, ENDO-ERN (European Reference Network on Rare Endocrine Conditions), FIN-00290 Helsinki, Finland

**Keywords:** Dxa, Men1, parathyroid-related disorders, Pth/vit d/fgf23, osteoporosis

## Abstract

Primary hyperparathyroidism (PHPT) associated with multiple endocrine neoplasia type 1 (MEN1) impairs bone mineral density and causes osteoporosis already in young patients. We aimed to investigate bone mineral density (BMD) in a contemporary cohort of patients with MEN1-related PHPT after long-term follow-up and compare these results with that of healthy controls. Thirty-five patients with genetically confirmed MEN1 were diagnosed with MEN1 at mean age 28.7 ± 13.6 years. Thirty-two (91.4%) underwent primary parathyroidectomy at mean age 33.3 ± 13.7 years; 12 had undergone at least 2 surgeries with on average 7.3 ± 5.9 years between the operations. BMD was assessed by DXA at the end of mean follow-up, 13.2 years after the primary parathyroidectomy and compared with that of 35 age- and gender-matched controls. More than 10 years after the first parathyroidectomy, mean BMD in patients with MEN1 is in the normal range. However, it is still significantly lower compared with healthy controls.

## Graphical Abstract

**Graphical Abstract:**
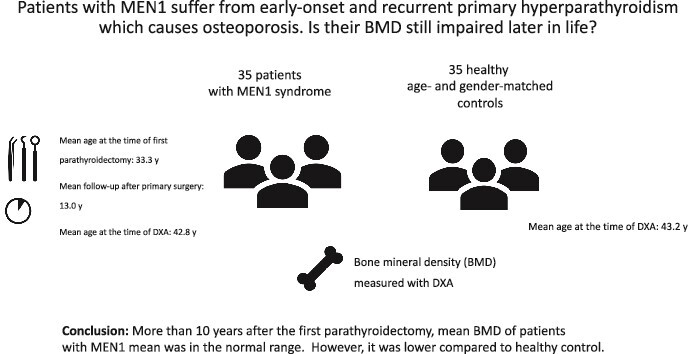

## Introduction

Primary hyperparathyroidism (PHPT) associated with multiple endocrine neoplasia type 1 (MEN1) impairs bone mineral density (BMD) and can lead to severe early-onset osteoporosis.^1^^,^^2^

While sporadic PHPT is characterized by single gland disease and late onset (>50-60 years), MEN1 predisposes to hyperplasia of all 4 parathyroid glands and early disease onset at a mean age of 20-25 years before peak bone mass is reached.^3^^,^^4^ Therefore, skeletal manifestations in MEN1-related PHPT are considered more severe than in sporadic PHPT.^5–7^ In the studies of Burgess et al.^7^ and Lourenco et al.^8^, untreated MEN1-related PHPT led to osteopenia in early adulthood and osteoporosis after the age of 50 years. Similar to sporadic PHPT, BMD increases after parathyroidectomy in MEN1 patients by 5%-8% in the lumbar spine (LS) and by 3%-6% in the femoral neck (FN), respectively.^5^^,^^7^ However, MEN1 is characterized by recurrent PHPT in 20%-60% of cases, depending on the extent of the surgical procedure and the length of follow-up.^9–11^ During their lifespans, MEN1 patients often suffer from recurrent or persistent PHPT and reoperations.

Contemporary MEN1 is often diagnosed at an early stage through genetic screening of the first-degree relatives of a known proband. This is in contrast to the situation before identification of the MEN1 gene in 1997,^12^ when diagnosis was based on the classical triad of 3 Ps: pituitary, parathyroid, and pancreatic tumors. The life-long, annual follow-up offered to mutation carriers has led to 10-15–year earlier diagnosis of MEN1 manifestations.^13^^,^^14^ Therefore, PHPT can be diagnosed at a very early stage in childhood or youth. However, it is not known whether there is still a concern regarding bone mass in contemporary adult patients with MEN1. Furthermore, it is not known whether the timing and extent of primary surgery for PHPT in MEN1 patients is appropriate, and how possible recurrences and further surgery affect long-term outcomes on bone metabolism.

Our aim was to study the long-term effects of early-onset PHPT on bone in a contemporary cohort of patients with MEN1 who had undergone their first parathyroidectomy at least 10 years earlier. As a result of genetic screening and regular follow-up, these patients were diagnosed with PHPT at a young age compared with older studies.^5^^,^^7^^,^^13^^,^^15^ At the end of the follow-up, BMD was compared with that of the age- and gender-matched controls. We present >10-year longitudinal follow-up data on PHPT and their management in MEN1, which, to our knowledge, represents the longest follow-up data reported so far.

## Materials and methods

### Study design

#### Patients

At the time of the study, there were 51 patients with MEN1 in follow-up at the Department of Endocrinology at Helsinki University Hospital, Helsinki, Finland, a reference center for rare endocrine conditions. The inclusion criteria of this study for the MEN1 patients were DXA performed between January 2018 and March 2022 and the diagnosis of MEN1 syndrome confirmed with genetic testing. Based on these criteria, 35 MEN1 patients (17 males and 18 females) were eligible for the study. Sixteen of the 51 patients were not suitable for the study or DXA was not performed (young age and no PHPT at the age of <20 years (3 patients), malignancy with bone metastases and antiresorptive bone medication (2 patients), death before DXA (1 patient), pregnancy including several pregnancies before initiation of the study (1 patient), long distance to hospital thus did not want to participate (5 patients), or DXA programmed but not performed (3 patients)). The data on the medical history of MEN1 manifestations were retrieved from our electronic patient records and analyzed anonymously. The diagnosis of PHPT was determined by hypercalcemia and by a PTH concentration either increased or in the upper range of normal. Age at the onset of PHPT was determined according to the first measurement of the increased serum ionized calcium concentration for each individual patient. A recurrence of PHPT was diagnosed when normocalcemia sustained over 6 months after surgery, and then developed to hypercalcemia with a serum PTH concentration either increased or in the upper range of normal.

The control group was collected from healthy individuals by matching age and gender with MEN1 patients. The male controls were, on average, older than their respective patients. Therefore, the female pairings were adjusted such that the female patients were matched with the younger controls. This was done to balance the average ages of the patient and control groups. This study was conducted according to the guidelines of the Declaration of Helsinki. The protocol was approved by the institutional board review of Helsinki University Hospital (HUS/115/2020).

### Bone densitometry measurements

The DXA of MEN1 patients was performed between January 2018 and March 2022. DXA and similar biochemical measurements of the controls were performed in 2017.

BMD was measured by DXA with Lunar Prodigy Advance (GE Medical Systems Corp.) in the control group and Hologic (Hologic, Inc., Waltham, MA, United States) in the patient group, except for 1 patient, whose BMD was measured with the above-mentioned Lunar equipment. All measurements were performed at the Diagnostic Center of Helsinki University Hospital. Absolute BMD values (g/cm^2^), T-scores, and Z-scores measured at 3 locations (LS, LS, L1-L4; FN; total hip) were collected retrospectively from medical records and chosen as primary endpoints.

### Biochemical measurements

We collected data on serum ionized calcium, alkaline phosphatase, phosphate, and 24-h urinary calcium concentrations from both the patient and control groups. Laboratory testing was performed during the same period as the DXA measurements. The mean absolute time difference between the DXA and laboratory exams was 0.04 ± 0.06 years, except for 24 h urinary calcium (a mean difference of 0.53 ± 0.88 years). MEN1 patients underwent annual laboratory testing according to the standard follow-up protocol. The test results, including biochemistry measured during previous follow-ups, were retrieved from individual medical records. All laboratory tests were performed at the central laboratory of Helsinki University Hospital, HUSLAB, using in-house methods. The reference ranges of the normal values are indicated in Table 1. For some patients, not all biochemistry was measured at the time of DXA, see Table 2.

**Table 1:** Clinical characteristics of MEN1 patients and controls.

	**MEN1 patients (*n* = 35)**	**Controls (*n* = 35)**	***p*-value**
**Age at DXA (y.o) (range)**	42.8 ± 15.7 (18-72)	43.2 ± 9.71 (31-60)	0.711
**Women % (n)**	0.51 (18)	0.51 (18)	1.000
**BMI (kg/m^2^)**	28.2 ± 5.62	25.3 ± 5.12	0.031
**Current smokers % (n)**	17 (6)	3 (1)	0.106
**Biochemical parameters**			
**Ca ion (mmol/L)**	1.31 ± 0.12 (1.02-1.46), *n* = 35	1.24 ± 0.03 (1.17-1.28)	0.001
**PTH (ng/l)**	96.6 ± 68.9 (11-268), *n* = 23	−	
**Phosphate (mmol/L)**	0.98 ± 0.30 (0.58-1.58), *n* = 15	0.98 ± 0.16 (0.69-1.36)	0.953
**Alkaline phosphatase (U/L)**	77.4 ± 19.8 (54-116), *n* = 16	65.9 ± 14.9 (42-96)	0.118
**25 OH D (nmol/L)**	70.0 ± 22.7 (26-114), *n* = 24	−	
**24-h urinary calcium (mmol/24 h)**	6.7 ± 4.9 (1.6-13.9), *n* = 22	2.3 ± 1.2 (0.5-5.4)	<0.001

Data are presented as numbers (percentages) or mean ± SD (range). Abbreviations: DXA = dual-energy X-ray absorptiometry, BMI = body mass index, Ca ion = serum ionized calcium, 25 OH D = serum 25-hydroxy D. Reference ranges: Serum ionized calcium 1.16*-*1.30 mmol/L, PTH 18*-*80 ng/L, phosphate 0.71*-*1.53 males 18*-*49 y, 0.71*-*1.23 males >50 y, 0.76*-*1.41 females >18 y mmol/L, alkaline phosphatase 35*-*105 U/L, 25 OH D > 50 nmol/L, 24-h urinary calcium 1.3*-*6.5 mmol/24 h

**Table 2:** Clinical characteristics of primary hyperparathyroidism and surgical treatment in MEN1 patients.

**Primary hyperparathyroidism**		
History of PHPT n (%)		35 (100)
**Age at the time of genetic testing of MEN1 (y)**		30.3 ± 16.3
Age at the diagnosis of PHPT (y)^*^		28.7 ± 13.6 (*n* = 25)
**Caion at the diagnosis of PHPT (mmol/L)**		1.42 ± 0.10 (*n* = 24)
**Persistent PHPT at the time of DXA n (%)**		22 (62.9)
**Ca ion in patients with PHPT at the time of DXA (mmol/L)**		1.38 ± 0.06
**Hypoparathyroidism at the time of DXA n (%)**		7 (20.0)
**Caion in patients with hypoparathyroidism at the time of DXA (mmol/L)**		1.12 ± 0.06
**Surgical treatment**		
**PHPT operated n (%)**		32 (91.4)
Age at the time of primary operation (y)^**^		33.3 ± 13.7
**Age at the time of second operation (y)**		32.1 ± 13.5 (*n* = 12)
**The number of operations for PHPT per patient**		1.5 ± 1.0
**The type of primary operation n (%)**		
	PPTX	15 (46.9)
	STPTX	5 (15.6)
	TPTX	12 (37.5)
**PHPT recurrences after primary operation n (%)**		21 (65.6)
**Recurrent PHPT in each type of primary operation n (%)**		
	PPTX	13 (86.7)
	STPTX	0 (0)
	TPTX	8 (66.7)
Time from primary operation to recurrence of PHPT (y)^***^		3.0 ± 4.8 (*n* = 15)
**Time between primary and second operation (y)**		7.3 ± 5.9
**Histology of removed gland(s) in primary operation n (%)**		
	Hyperplasia	19 (59.4)
	Adenoma	3 (9.4)
	Carcinoma	0 (0)
	Pathology report unavailable	10 (31.3)

Abbreviations: y = years, PHPT = primary hyperparathyroidism, Ca ion = serum ionized calcium, reference range 1.15-1.30 mmol/L, DXA = dual energy X-ray absorptiometry. PPTX = partial parathyroidectomy (removal of parathyroids up to 3 glands); STPTX = subtotal parathyroidectomy (removal of 3 parathyroids and a part of the fourth gland); TPTX = total parathyroidectomy (all parathyroids removed with normal parathyroid tissue auto-transplanted into the forearm).

^*^Range 11-59 years.

^**^Determined by the first elevated serum ionized calcium value measured from each patient.

^***^Determined by the onset of hypercalcemia after the operation. Data are presented as mean ± SD.

### Surgical treatment

Surgical interventions were performed in 3 university and 3 central hospitals. The data regarding the type of surgery and pathological diagnosis were retrieved from individual medical records.

MEN1 patients who had undergone surgical treatment for PHPT were classified into 3 groups according to the approach of their primary surgery: partial parathyroidectomy (PPTX; the removal of parathyroids, up to 3 glands), subtotal parathyroidectomy (STPTX; the removal of 3 parathyroids and part of the fourth gland), and total parathyroidectomy (TPTX; the removal of all parathyroids with normal parathyroid tissue auto-transplanted into forearm).

### Statistics

Data are described as mean ± standard deviation (SD) for continuous variables or as proportions and absolute frequencies for categorical variables in MEN1 patients and controls. We assessed normal distributions for continuous variables in both groups with the Shapiro–Wilk test, and when considered normally distributed (*p*-value >0.05), groups were compared using Student t-test. For continuous but not normally distributed variables, the groups were analyzed using the Mann–Whitney U test. For categorical variables, Fisher test was used. Finally, a linear regression model was performed to assess the impact of different variables on BMD in MEN1 patients. In all statistics, the level of significance was set at a *p*-value <0.05. All statistical analyses were performed using SPSS version 25.

## Results

### Baseline clinical characteristics and biochemistry of MEN1 and control subjects

The clinical characteristics and biochemical parameters of the MEN1 patients and controls are shown in Table 1. There were no significant differences in age between the total patient and control groups (42.8 ± 15.7 vs. 43.2 ± 9.7 years, *p* = .711) or between the subgroups of women and men. Body mass index (BMI) was higher in the patient group (28.2 ± 5.6 vs. 25.3 ± 5.1 kg/m^2^, *p* = .031).

The biochemistry of the 2 groups is presented in Table 1. Significant differences between the study and control groups were found for serum ionized calcium concentrations (1.31 ± 0.12 mmol/L vs. 1.24 ± 0.03 mmol/L, *p* = .001) and 24 h urinary calcium secretion (6.67 ± 3.99 vs. 2.33 ± 1.19 mmol/24 h, *p* < .001, respectively).

### Clinical course and surgical treatment of PHPT in patients with MEN1

The clinical characteristics of PHPT and parathyroidectomies performed among MEN1 patients are shown in Table 2. All MEN1 patients had or had previously suffered from PHPT. At the onset of PHPT, the mean age of the patients was 28.7 ± 13.6 years, and the serum ionized calcium was 1.42 ± 0.10 mmol/L. The mean age at genetic diagnosis of MEN1 was 30.3 ± 16.3 years.

**Figure 1:**
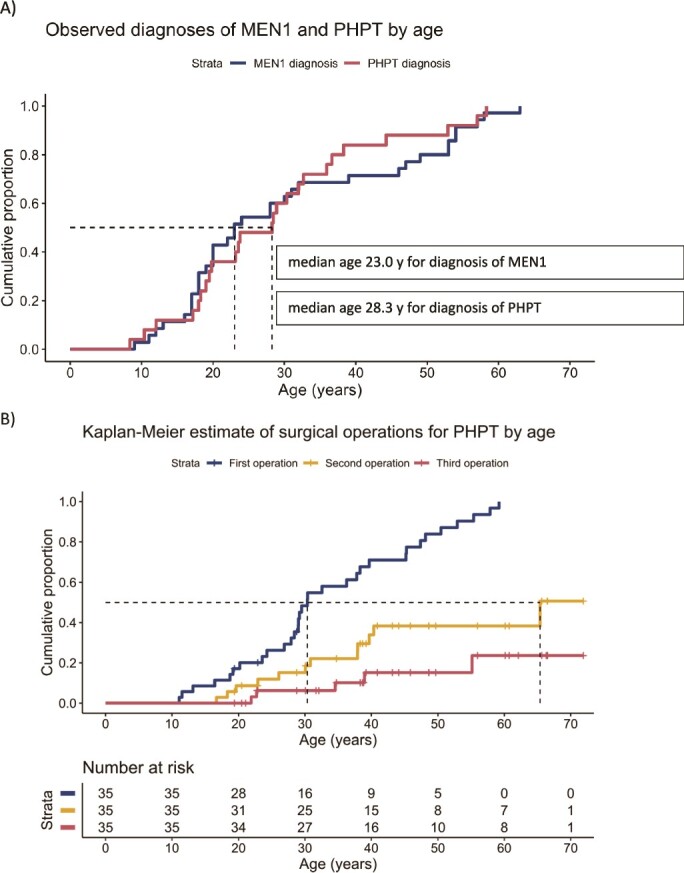
A) Age at genetic diagnosis of MEN1 and age at diagnosis of PHPT in patients with MEN1. The median age at which half of the patients were diagnosed was 23.0 years for MEN1 and 28.3 years for PHPT (dashed lines). B) Kaplan–Meier estimate of surgical operations for PHPT in patients with MEN1. The dashed line denotes the estimate for the age at which half of the patients have received surgery.

The comparison of ages at diagnosis of PHPT and at confirmation of MEN1 diagnosis is shown in Figure 1A.

Thirty-two out of 35 patients (91.4%) had undergone at least 1 parathyroid operation, and the mean age at the primary operation was 33.3 ± 13.7 years. The number of PHPT operations was 1.5 ± 1.0 (range 0-5), and 12 patients had at least 2 operations, with an average of 7.3 ± 5.9 years between the 2 (Figure 1B). After a mean follow-up of 13.0 ± 10.8 years (range 0.4-42.2 years) after primary surgery, 21 (65.6%) of the operated patients had persistent or recurrent PHPT characterized at the time of DXA by mild hypercalcemia (mean serum ionized calcium 1.34 ± 0.12 mmol/L). On average, these patients developed hypercalcemia at 3.0 ± 4.8 years (*n* = 15) after surgery, and 3 of them had persistent PHPT.

The surgical approach of the primary operation was PPTX in 15, STPTX in 5, and TPTX in 12 patients. The type of primary operation among the 7 patients with hypoparathyroidism at the time of DXA was PPTX in 3, STPTX in 2, and TPTX in 2 patients. However, 2 of the patients whose primary surgery was PPTX needed a second operation, after which hypoparathyroidism occurred. Of 12 patients classified as TPTX, 4 patients operated on in 1999-2005 underwent TPTX with part of the fourth gland transplanted into the forearm in 6 to 20 pieces. Three of these patients later developed hypercalcemia, and some of the transplants were removed from the forearm; 1 of them was reoperated up to 4 times and was still hypercalcemic at the time of DXA. Histopathological diagnosis was parathyroid hyperplasia in 59.4% and adenoma in 9.4%; no one had parathyroid carcinoma. The data were not available in 31.3% of the cases.

### Other MEN1 manifestations with possible negative effects on BMD

Nine of the 35 patients (25.7%) also had another MEN1 manifestation that possibly affected the bone. Three (8.6%) MEN1 patients had a history of hypercortisolism: 1 patient suffered from bilateral adrenal gland hyperplasia, 1 had ACTH-secreting neuroendocrine tumor in the pancreas and nodular cortical hyperplasia of the adrenal glands, and 1 had ACTH-secreting pituitary adenoma. At the time of DXA, 1 patient was surgically cured, and 2 patients still suffered from mild hypercortisolism (including the patient that was operated on). Seven (20.0%) MEN1 patients (6 females and 1 male) were diagnosed with prolactinoma. All had received cabergoline treatment. Three women still had elevated serum prolactin concentrations at the time of DXA (947 ± 140 mU/L, reference range 102-496 mU/L for women).

### Bone mineral density

In 28 of 35 patients with DXA measured after the primary operation, the time between primary surgery and DXA was 13.2 ± 10.0 years. Of the 35 patients, 4 underwent primary surgery after DXA, and 3 others had mild PHPT and were not yet planned for surgery. At the time of DXA, 22 patients (62.9%) had mild persisting hyperparathyroidism (mean serum ionized calcium 1.38 ± 0.06 mmol/L), 7 patients (20.0%) suffered from postoperative hypoparathyroidism (mean serum ionized calcium 1.12 ± 0.06 mmol/L), and 6 patients (17.1%) had normal parathyroid hormone function (ie, normocalcemia without calcium or alfacalcidol supplements).

**Figure 2:**
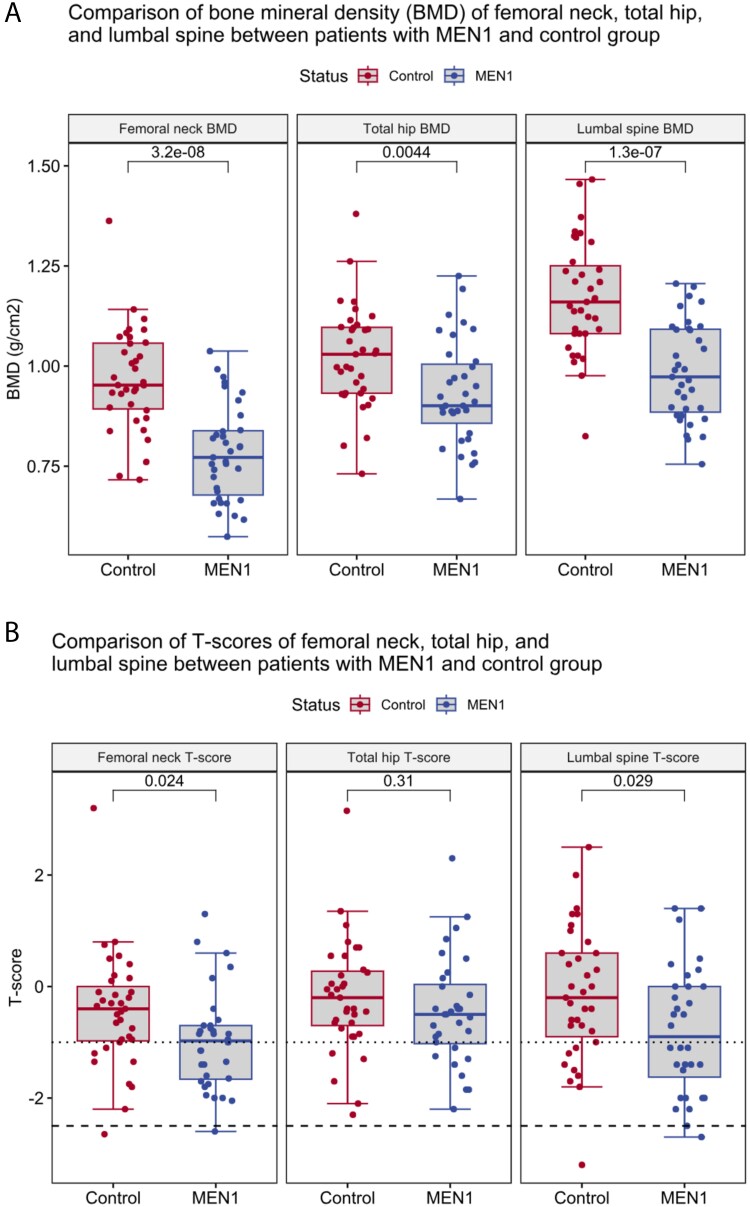
Boxplots comparing BMD in the femoral neck, total hip, and lumbar spine in patients with MEN1 and healthy controls. *p*-value for comparison between groups is shown above the plots.

The results from DXA are shown in Table 3 and Figure 2. Absolute values, expressed as g/cm^2^, were significantly lower in the patient group than in the control group at all 3 locations. The mean BMD was lower in patients group than in the control group at all 3 sites (LS BMD 0.986 ± 0.123 g/cm^2^ vs. 1.172 ± 0.139 g/cm^2^ (*p* < .001), BMD 0.782 ± 0.119 g/cm^2^ vs. 0.967 ± 0.129 g/cm^2^ (*p* < .001), Total hip BMD 0.931 ± 0.130 g/cm^2^ vs. 1.022 ± 0.128 g/cm^2^ (*p* = .004), respectively). There were also significant differences between groups in T-scores at LS (-0.788 ± 1.140 vs. -0.151 ± 1.194, *p* = .030) and at FN (-0.988 ± 0.892 vs. -0.447 ± 1.025, *p* = .012), both indicating lower BMD in the patient group.

**Table 3:** Bone mineral density in MEN1 patients compared with healthy age- and gender-matched controls.

	**MEN1 patients (*n* = 35)**	**Controls (*n* = 35)**	***p*-value**
**Lumbar spine**			
**BMD (g/cm^2^)**	**0.986 (0.123)**	**1.172 (0.139)**	**<0.001**
**T-score**	**−0.79 (1.14)**	**−0.15 (1.19)**	**0.03**
**Z-score**	−0.29 (1.14)	−0.10 (1.18)	0.49
**Femoral neck**			
**BMD (g/cm^2^)**	**0.782 (0.119)**	**0.967 (0.129)**	**<0.001**
**T-score**	**−0.99 (0.89)**	**−0.45 (1.03)**	**0.012**
**Z-score**	−0.37 (0.67)	−0.19 (0.98)	0.356
**Total hip**			
**BMD (g/cm^2^)**	**0.931 (0.130)**	**1.022 (0.128)**	**0.004**
**T-score**	−0.44 (0.98)	−0.19 (1.01)	0.309
**Z-score**	−0.10 (0.80)	−0.04 (0.95)	0.778

Abbreviation: BMD = bone mineral density. Data are presented as mean ± SD. Bold font indicates statistical significance.

### DXA findings in MEN1 according to gender

BMD results according to gender are shown separately in Table 4. Mean age of women was 47.3 years (range 21-72). At the time of DXA, 10 of the 18 women were premenopausal and 8 were postmenopausal.

**Table 4:** Bone mineral density according to gender in MEN1 patients and healthy controls.

	Females			Males		
	**MEN1 patients**	**Controls**	***p*-value**	**MEN1 patients**	**Controls**	***p*-value**
**Lumbar spine**						
**BMD (g/cm^2^)**	**0.978 (0.126)**	**1.179 (0.117)**	**<0.001**	**0.995 (0.122)**	**1.164 (0.163)**	**0.002**
**T-score**	−0.62 (1.25)	0.09 (0.99)	0.066	−1.00 (1.00)	−0.41 (1.37)	0.185
**Z-score**	0.04 (1.12)	0.13 (0.80)	0.779	−0.64 (1.10)	−0.35 (1.46)	0.530
**Femoral neck**						
**BMD (g/cm^2^)**	**0.752 (0.114)**	**0.939 (0.143)**	**<0.001**	**0.814 (0.119)**	**0.998 (0.108)**	**<0.001**
**T-score**	−0.96 (0.95)	−0.34 (1.19)	0.062	−1.09 (0.85)	−0.56 (0.83)	0.143
**Z-score**	−0.32 (0.64)	−0.23 (1.06)	0.597	−0.43 (0.72)	−0.14 (0.90)	0.301
**Total hip**						
**BMD (g/cm^2^)**	0.906 (0.133)	0.992 (0.142)	0.072	**0.957 (0.124)**	**1.053 (0.106)**	**0.021**
**T-score**	−0.34 (1.11)	−0.12 (1.19)	0.560	−0.58 (0.80)	−0.28 (0.82)	0.316
**Z-score**	0.13 (0.81)	−0.07 (1.02)	0.530	−0.32 (0.74)	0.00 (0.91)	0.268

Abbreviation: BMD = bone mineral density. Data are presented as mean (SD). Bold value indicates statistical significance.

For females, mean LS absolute BMD, T-score and Z-score were 0.978 ± 0.126 g/cm2 (*p* < .001), -0.622 ± 1.245 SD (*p* = .066), 0.041 ± 1.116 SD (*p* = .779), respectively, *p*-values compared with controls. In FN, mean BMD, T-score, and Z-score were 0.752 ± 0.114 g/cm2 (*p* < .001), -0.964 ± 0.949 SD (*p* = .062), and -0.318 ± 0.635 SD (*p* = .597) and total hip mean BMD, T-score, and Z-scores were 0.906 ± 0.133 g/cm2 (*p* = .072), -0.342 ± 1.106 SD (*p* = .560), and 0.127 ± 0.811 SD (*p* = .530), respectively, compared with controls.

Similarly, mean age of men was 36.7 years (range 18-70); 3 of them were over 50 years of age. For males, LS mean BMD, T-score, and Z-score were 0.995 ± 0.122 g/cm2 (*p* = .002), -1.000 ± 0.992 SD (*p* = .185), and -0.629 ± 1.101 SD (*p* = .530). In FN, mean BMD, T-score, and Z-score were 0.814 ± 0.119 g/cm2 (*p* < .001), -1.02 ± 0.848 SD (*p* = .143), and -0.429 ± 0.721 SD (*p* = .301). For total hip, mean BMD, T-score, and Z-score were 0.957 ± 0.124 g/cm2 (*p* = .021), -0.575 ± 0.797 SD (*p* = .316), and -0.318 ± 0.743 SD (*p* = .268).

There were no significant differences between the BMD in controlled vs. uncontrolled PHPT (ie, in those with cured vs. persistent or recurrent PHPT) in the T- or Z-scores of these 2 groups (data not shown).

A linear regression model was used in the patient group to study the association of variables (BMI, age at diagnosis, age at primary operation, presence of persistent or recurrent PHPT at the time of DXA, presence of hypoparathyroidism, serum ionized and 24 h urinary calcium, PTH, plasma phosphate, alkaline phosphatase, 25-OH D, the number of operations for PHPT, and smoking), with BMD at all 3 locations. The model did not show any significant correlations between BMD and the suggested variables, except for age at DXA acting as a predictive variable for total hip T-score (*p* = .031).

## Discussion

In this retrospective longitudinal study, encompassing >10-years’ mean follow-up of PHPT in MEN1, we compare BMD of MEN1 patients with healthy controls and describe the clinical course of PHPT in a contemporary MEN1 cohort. To our knowledge, this is the first study evaluating the long-term outcomes of parathyroidectomy on bone health in MEN1, including a comparison of BMD of MEN1 patients with that of age- and gender-matched healthy adults with confirmed normal calcium metabolism.

The main finding was that the BMD in MEN1 patients after long-term follow-up was within the normal reference range. For male patients, the mean BMD T-scores in LS and FN were -1.0 SD, but the mean Z-scores were still in the normal range. However, compared with the controls, the absolute BMD values in all measured sites and the T-scores in LS and FN were still significantly lower. These differences remained significant when studied separately in women and men.

In addition, we provide in-depth longitudinal data on the types of surgeries performed for PHPT in MEN1 and their outcomes.

In this study, PHPT was diagnosed at a mean age of 28.7 years as an early manifestation of MEN1. In previous studies showing decreased BMD in MEN1, the mean age at the diagnosis of PHPT was 38.9 years^8^ and 47.1 years for probands and 40 years for their relatives, which were diagnosed during the study.^15^ In 2 more recent MEN1 cohorts, the mean age at diagnosis of PHPT was 34 years.^2^^,^^16^ Before parathyroidectomy, MEN1 patients with an average age of 42 years had T-scores of -1.6 and -1.7 SD in LS and FN, respectively.^17^ Instead, in a more recent MEN1 cohort with a mean age of 34.2 years, BMD was not impaired before initial parathyroidectomy.^16^ These results suggest that early diagnosis of PHPT can prevent bone deterioration, and higher age at diagnosis of MEN1-related PHPT can explain the low BMD of earlier studies.

In our study patients, the genetic diagnosis of MEN1 was confirmed at a younger age (a mean age of 30.3 years), compared with that of 42–47 years for probands and 34–37 years for their relatives reported in 2 large MEN1 database studies.^13^^,^^14^ Of note, over 40% of our patients were diagnosed by the age of 20 (Figure 1A). The Dutch MEN1 database^9^ reported PHPT in 20.3% and 39.5% of MEN1 patients diagnosed between 10-20 and 21-30 years of age, respectively. Earlier diagnosis of MEN1 is known to decrease both overall morbidity and mortality in MEN1 patients^18^^,^^19^ and, according to this study, seems also to prevent severe bone manifestation of PHPT.

In this study, the average T- and Z-scores of BMD measured at all 3 sites were within the reference range. The time interval of 13.2 years between the first parathyroidectomy and DXA measurement in MEN1 patients who underwent parathyroidectomy (80% of the cohort) was sufficiently long to show the long-term impact of MEN1-related PHPT and to detect possible increases in BMD after parathyroidectomies. The fact that BMD was not assessed by DXA at the time of the first parathyroidectomy can be regarded as a limitation of this study. Bone manifestations of MEN1-related PHPT are considered more severe than in sporadic PHPT,^1^^,^^2^^,^^20^ and Burgess et al.^7^ and Lourenco et al.^8^ reported osteopenia or osteoporosis before the first parathyroidectomy.^21^ Two smaller, short-term studies including a total of 21 patients previously reported the outcome of primary parathyroidectomy on BMD in MEN1, with 5.2%-8.4% improvements in LS and 3.2%-6.9% improvements in FN 15-18 months after surgery,^5^^,^^7^ similarly to sporadic PHPT.^22^ The bone microarchitecture in cortical and trabecular sites seems disturbed in MEN1-related, as in sporadic, PHPT.^23^

Current guidelines for PHPT recommend parathyroidectomy for all patients diagnosed before the age of 50 years^24^ and do not differentiate sporadic from MEN1-related PHPT. Although the conventional criteria for surgery are fulfilled due to the young age of patients with MEN1, the appropriate timing of primary parathyroidectomy in these often-asymptomatic patients is unclear, and specific recommendations are lacking. However, our results show that a near-normal, long-term BMD can be achieved even though asymptomatic patients with PHPT are not operated on during adolescence but at the age of 30.

In this study, 91.4% of the patients had undergone parathyroidectomy, and PHPT recurred in 65.6% of these patients during the 13 years of follow-up. Previous studies report a recurrence rate of 20%-60%,^7^^,^^16^ depending on the length of follow-up and extent of surgery.^16^ Although recurrent PHPT can decrease BMD, we demonstrate that this was not the case in the present cohort of MEN1 patients, as BMD after long-term follow-up was within normal reference ranges, suggesting successful management of PHPT.

According to current guidelines, parathyroidectomy of MEN1-related PHPT should include removal of 3-3.5 parathyroid glands.^25^^,^^26^ In this study, recurred cases had either undergone PPTX (62%; removal of up to 3 glands) or TPTX (38%), with autotransplantation as the primary surgery. Interestingly, there were no recurrences after subtotal PTX (removal of 3.5 glands) was performed on 15.6% of the patients. Reoperation exposes patients to surgical complications, especially to hypoparathyroidism.^27^^,^^28^ As TPTX is related to the highest risk of permanent hypoparathyroidism,^11^^,^^29^^,^^30^ the current recommendations favor subtotal surgery for MEN1-related PHPT.^31^^,^^32^

In addition, other MEN1 manifestations, such as hypercortisolism and hyperprolactinemia, can reduce BMD. Prolactinoma is present in one-third of MEN1 children under 16 years of age.^3^

In this study, 7 patients were treated for prolactinoma, and 3 had a past history of hypercortisolism.

The strength of our study is the length of follow-up and a comparison with age- and gender-matched subjects with normal calcium metabolism. We provide detailed information on the medical history and biochemistry data retrieved from the medical records, as well as the confirmed MEN1 diagnosis with genetic testing. Over half of our patients were diagnosed with MEN1 by the age of 20-25. The limitation of our study is the lack of fracture data.

## Conclusion

More than 10 years after the first parathyroidectomy, BMD was still within the reference range for MEN1-related PHPT. This can be explained by the lower mean age at genetic confirmation of MEN1 and at diagnosis of PHPT compared with previous patients diagnosed in earlier decades. However, BMD was still significantly impaired compared with the healthy controls. These real-life data encourage continuing the surveillance of asymptomatic PHPT in adolescents with MEN1 and proceeding to surgery when the serum calcium concentration approaches the criteria for surgery.

## Data Availability

The collected data used for the analysis may enable identification of a patient. Therefore the detailed data used for the statistical analysis are available only for reviewers on request.
